# *Piscirickettsia salmonis* shedding and tissue burden, and hematological responses during cohabitation infections in chum *Oncorhynchus keta*, pink *O*. *gorbuscha* and Atlantic salmon *Salmo salar*

**DOI:** 10.1371/journal.pone.0248098

**Published:** 2021-03-05

**Authors:** Amy Long, Simon R. M. Jones

**Affiliations:** Pacific Biological Station, Fisheries and Oceans Canada, Nanaimo, British Columbia, Canada; University of Idaho, UNITED STATES

## Abstract

**Background:**

Salmonid rickettsial septicemia is an emergent and geographically widespread disease of marine-farmed salmonids caused by infection with the water-borne bacterium *Piscirickettsia salmonis*. Very little is known about the route, timing, or magnitude of bacterial shedding from infected fish.

**Methodology/principal findings:**

A cohabitation challenge model was used to assess shedding from chum *Oncorhynchus keta*, pink *O*. *gorbuscha* and Atlantic salmon *Salmo salar*. Infections in donor fish were established by intraperitoneal injection of *P*. *salmonis*. Naïve recipients were cohabitated with donor fish after which cumulative percent morbidity and mortality (CMM) was monitored, and bacterial burdens in kidney and in tank water were measured by qPCR. All donor fish died with mean days-to-death (MDD) among species ranging from 17.5 to 23.9. Among recipients, CMM ranged from 42.7% to 77.8% and MDD ranged from 49.7 to 56.4. In each trial, two peaks of bacterial DNA concentrations in tank water closely aligned with the MDD values of donor and recipient fish. Bacterial tissue burden and shedding rate, and plasma physiological parameters were obtained from individual donors and recipients. Statistically significant positive correlations between the shedding rate and *P*. *salmonis* kidney burden were measured in donor pink and in donor and recipient chum salmon, but not in donor or recipient Atlantic salmon. In Atlantic salmon, there was a negative correlation between kidney bacterial burden and hematocrit, plasma Ca^++^ and Mg^++^ values, whereas in infected chum salmon the correlation was positive for Na^+^ and Cl^-^ and negative for glucose.

**Conclusions:**

A dependency of bacterial shedding on species-specific patterns of pathogenesis was suggested. The coincidence of bacterial shedding with mortality will inform pathogen transmission models.

## Introduction

The Gram-negative bacterium *Piscirickettsia salmonis* causes salmonid rickettsial septicemia (SRS) or piscirickettsiosis in salmonids farmed in marine open netpens [1, 2]. SRS is a leading cause of mortality associated with infectious disease in farmed salmon in Chile [2], and presents a significant economic challenge to the industry in that country [3]. The disease also occurs sporadically in farmed salmon in eastern and western Canada, Norway, Scotland and Ireland where its impact has been relatively minor [4–8].

Evidence for waterborne transmission of *P*. *salmonis* comes from epidemiological and laboratory studies using cohabitation or immersion modes of exposure [9–14]. Contact among fish or osmotic protection of *P*. *salmonis* within sloughed fish tissue or mucous facilitates laboratory transmission of the bacterium and promotes its viability, particularly in freshwater [11, 15]. The lesions reported from the skin and gut of infected fish suggest these are sites of microbial shedding, and *P*. *salmonis* may also be shed via bile, feces and urine [2]. Long et al. [14] provided the first quantitative estimates of *P*. *salmonis* shedding from Atlantic (*Salmo salar*) and sockeye (*Oncorhynchus nerka*) salmon. However, despite their importance in understanding pathogenesis and the formulation of epidemiological models, the route, timing, and magnitude of *P*. *salmonis* shedding from individual fish are poorly understood.

The present study reports patterns of *P*. *salmonis* shedding during laboratory cohabitation exposures with chum (*O*. *keta*), pink (*O*. *gorbuscha*) and Atlantic salmon. The null hypotheses that shedding rates of *P*. *salmonis* are uniform among exposed individuals, that shedding rates of *P*. *salmonis* are unrelated to bacterial burden in kidney, and that perturbations in plasma physiology parameters were unrelated to bacterial burden in kidney were tested using data from individual Atlantic and chum salmon.

## Materials and methods

### Fish sources, maintenance and welfare

Pink salmon obtained from Quinsam River hatchery in 2017 (brood year 2016) and 2018 (brood year 2017) were transitioned to seawater 17 d after arrival at the Pacific Biological Station (PBS; Nanaimo, BC). Chum salmon obtained from the Nanaimo River hatchery were transitioned to seawater 10 months prior to challenge. Juvenile Atlantic salmon obtained from a commercial hatchery on Vancouver Island, were reared in brackish water, and transitioned to seawater 8 wk prior to challenge. In all trials, fish were held in 400 L tanks provided with 15°C sand-filtered and UV-treated flow-through seawater (7–8 L/min) under a 12 hr:12 hr photoperiod. The fish were fed a commercial diet (EWOS Canada) at a daily rate of 1% total biomass.

Fish were maintained in accordance with recommendations of the Canadian Council on Animal Care and approved by the Pacific Region Animal Care Committee (AUP 18-021A1). All experimentation was conducted at the PBS by appropriately trained staff. There was no history of infectious disease in fish populations prior to their arrival at PBS. Prior to challenge, five individuals from each population tested negative for *P*. *salmonis* by quantitative PCR and bacteriology. Following challenge, fish were monitored two- to three-times daily for mortality and for clinical signs of SRS (i.e., severe signs of lethargy and/or hyperventilation and/or uncoordinated swimming, external gross lesions). Moribund fish showing clinical signs of SRS were euthanized by immersion in tricaine methanesulfonate (Syndel Canada) (MS-222; 200 mg/L). The number of dead and moribund fish removed daily contributed to the cumulative mortality and morbidity (CMM) for each tank. Sedation, anesthesia and euthanasia of salmon were employed as required, as described in the relevant sections.

### Bacterial culture

*Piscirickettsia salmonis* (isolate SR-1) was maintained in culture as previously described [14]. An injection inoculum was prepared from infected CHSE-214 [16] cells showing confluent cytopathic effect. Cultures were harvested and 1.25 mL of the harvested cells and tissue culture media was transferred to 50 mL of BM4 liquid medium [17] and incubated at 15°C for 48 hr with gentle stirring. The concentration of colony-forming units (CFU/mL) in the inoculum was determined by spread-plating 100 μL each from a series of dilutions in BM4 onto BCG agar [18] in duplicate. Plates were incubated at 15°C for 14–21 d and the number of colonies counted.

### Experimental design

Table 1 summarizes key elements of the experimental design. In the cohabitation trials 1, 2, and 4, mortality and morbidity were monitored in the cohabitation tanks and water samples were collected from these tanks to estimate bacterial burden. Bacterial shedding, kidney tissue burden, and physiological measurements were collected from individual donors and recipients in separate tanks. Trial 3 was conducted to measure individual shedding rates and bacterial burdens in donor pink salmon without naïve recipients and fish were monitored for 13 days post-injection (dpi).

**Table 1:** Design parameters for *Piscirickettsia salmonis* trials.

Trial	Date (M/Y)	Species	Number of tanks per treatment^1^	Mean weight (g)	Fish per tank^2^	Stock density (kg/m^3^)	Injection dose (CFU/g)
1	08/18	Pink	1C, 3H	217	10	10.9	2.0 x 10^2^
2	01/19	Chum	1C, 3H, 1D, 1R	52	25	6.5	9.6 x 10^2^
3	03/19	Pink	1D	177	24	10.6	1.1 x 10^3^
4	06/19	Atlantic	1C, 3H, 1D, 1R	151	21	15.6	6.5 x 10^2^

^1^C - negative control; H—cohabitation; D–individual donor sample; R–individual recipient sample (includes an equal number of donor fish).

^2^Number of donor, recipient, sample, or control fish in respective tanks.

### Cohabitation challenge

Prior to administering the intraperitoneal (ip) injection inoculum, water flow to each tank was stopped and 0.25 mg/L metomidate hydrochloride (Aquacalm; Syndel Canada) was added to the water as a sedative. After 15 min, fish were transferred to a 20 L temporary tank with aerated seawater containing Aquacalm. Groups of three sedated fish were then added to a 20 L tank containing 50 mg/L MS-222. Once anesthetized, each fish was removed, adipose fin clipped for identification as a donor, ip-injected with the inoculum, and returned to the original tank. Fish in the negative control tank were administered an ip injection of sterile BM4. Approximately 24 hr later, an equivalent number of naïve recipients were added to the cohabitation tanks (Table 1).

Trial 1 was a preliminary trial to optimize the water sampling methodology and to confirm pathogenicity of *P*. *salmonis* SR-1, and mortalities were not screened for *P*. *salmonis*. In Trials 2 and 4, dead, moribund, and surviving fish were stored frozen prior to bacterial screening by qPCR. In both trials, kidney samples were collected from 10% of donor mortalities, 20% of recipient mortalities, and from 100% of survivors, and preserved in 95% ethanol. Fish were monitored in Trial 1 for 59 dpi, and in Trials 2 and 4 for 65 dpi.

### Tank water sampling

Water samples were collected as described in [14] to estimate bacterial burden during cohabitation trials. In Trial 1, samples were collected every 3^rd^ day from 3 to 15 dpi, on 21, 25, and 28 dpi and every 3^rd^ day from 45 to 60 dpi. In Trial 2, samples were collected every 2^nd^ day from 9 to 23 dpi, every 4^th^ day from 29 to 43 dpi and every 2^nd^ day from 43 to 63 dpi. In Trial 4, samples were collected every 2^nd^ day from 13 to 65 dpi.

### Individual fish samples

Bacterial shedding rates were estimated from individual donors and recipients, and tissue and blood samples were collected from these fish in Trials 2 (chum) and 4 (Atlantic). Sampling times were chosen to coincide with pre-morbidity and early morbidity phases of the infection. In Trial 2, donors (n = 10) were sampled at 10 and 15 dpi, and recipients (n = 10) at 31 and 53 dpi. Negative control fish were sampled at each sampling time (n = 5). In Trial 4, donors were sampled at 10 (n = 5) and 17 dpi (n = 12), and recipients at 38 (n = 5) and 46 dpi (n = 12). Negative controls were sampled at each sample time (n = 5). In Trial 3 (pink), donors were sampled for individual shedding rates and tissue bacterial burdens at 8 (n = 10) and 13 (n = 10) dpi.

To estimate the individual shedding rate, water flow to the tank was stopped and 0.25 mg/L Aquacalm added. After 15 min, fish were individually netted into separate buckets containing approximately 5 L seawater. Water samples of 40 mL taken immediately (T_0_) and after 30 min (T_30_) were stored on ice and once sampling was complete, frozen at -80°C. The fish were then killed by the addition of 200 mg/L MS-222 to each bucket. Fork length and weight measurements were obtained for each fish. Blood was collected for measurement of hematocrit and analysis of physiological parameters in plasma, as described in Long et al. [19].

### DNA extraction and quantitative PCR

DNA was extracted from thawed water samples and from ethanol-preserved kidney as described in [14]. Bacterial burdens in the water and tissue samples were determined using a qPCR assay designed for the *P*. *salmonis* 23S gene [20, 21]. The limit of detection (LOD) for this assay is 5 copies/reaction (c/rxn) or 1 genome equivalent (GEq) [21]. The limit of quantification (LOQ) for this assay was determined using a curve-fitting modeling approach [22] to be 22 c/rxn. Detection of the bacterium in this study refers only to those samples in which bacterial DNA concentration was at or above the LOQ.

### Data analysis

Mean days-to-death (MDD) was calculated as the arithmetic mean of the number days between injection and individual mortality (or onset of morbidity) in a treatment group. Tank bacterial burdens in GEq/mL were standardized to the number of fish (live and dead) in the tank at time of sampling. The median Geq/mL/fish and its interquartile range (IQR) among tanks was calculated for each sample date. Total bacteria shed by donors and recipients in Trials 2 and 4 was estimated by area under the curve (AUC) from plots of Geq/mL/fish over time, as described earlier [23]. Individual shedding rates (S_i_) were calculated using the formula: S_i_ = (T_30_ GEq/mL–T_0_ GEq/mL) ÷ (W_i_ x T), where W_i_ is individual fish weight (g) and T is shedding time (min).

Bacterial burden in kidney was log_10_ transformed prior to analysis. Differences in log_10_ bacterial burden values between sampling points were evaluated using a Wilcoxon test. The statistical significance of differences in physiological measurements between negative control and infected fish at each sample time was assessed using Student’s t-test. Pearson’s Product-Moment Correlation (*r*) was determined at each sample time for bacterial burden in kidney and shedding rate or physiological parameters. All analyses were done in R version 3.6.1 and results were considered statistically significant if *P* ≤ 0.05.

## Results

### Morbidity, mortality and confirmation of infection

In all species, morbid and dead donor and recipient fish exhibited external lesions including scale loss, skin ulcers with peripheral darkening, petechial hemorrhaging, and areas of redness. Ulcer severity ranged from shallow (dermal layer still intact) to deep (underlying muscle exposed). The mean cumulative percent morbidity and mortality (CMM) of donor fish in all four trials was 100%.

**Fig 1:**
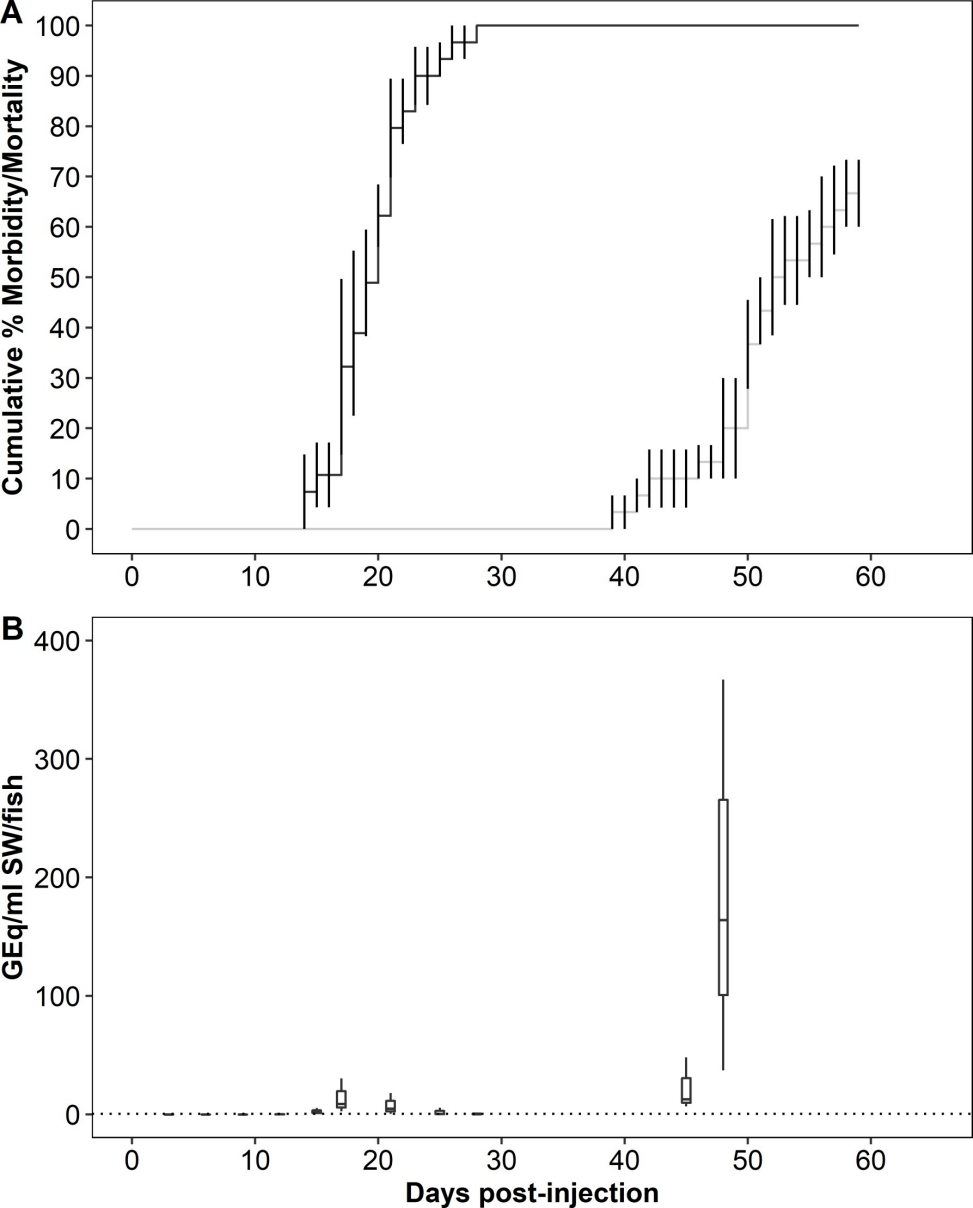
Mortality and bacterial burden in water during *Piscirickettsia salmonis* cohabitation challenge in pink salmon (Trial 1). (A) Cumulative mean percent morbidity and mortality for donor (black) and recipient (gray) populations, error bars denote standard error. (B) *P*. *salmonis* genome equivalents in tank water. Data are presented in box plots in which the inner horizontal line is the median, and the upper and lower boundaries of the box correspond to the first and third quartiles. The upper and lower whiskers denote the largest and smallest values no further than 1.5 times the inter-quartile range. The dotted horizontal line denotes assay limit of quantification.

In Trial 1 (pink cohabitation), the mean (± standard error) CMM among recipients was 66.7 ± 6.7 (Fig 1A), and the mean days to death (MDD) were 19.7 (donor) and 50.0 (recipient).

**Fig 2:**
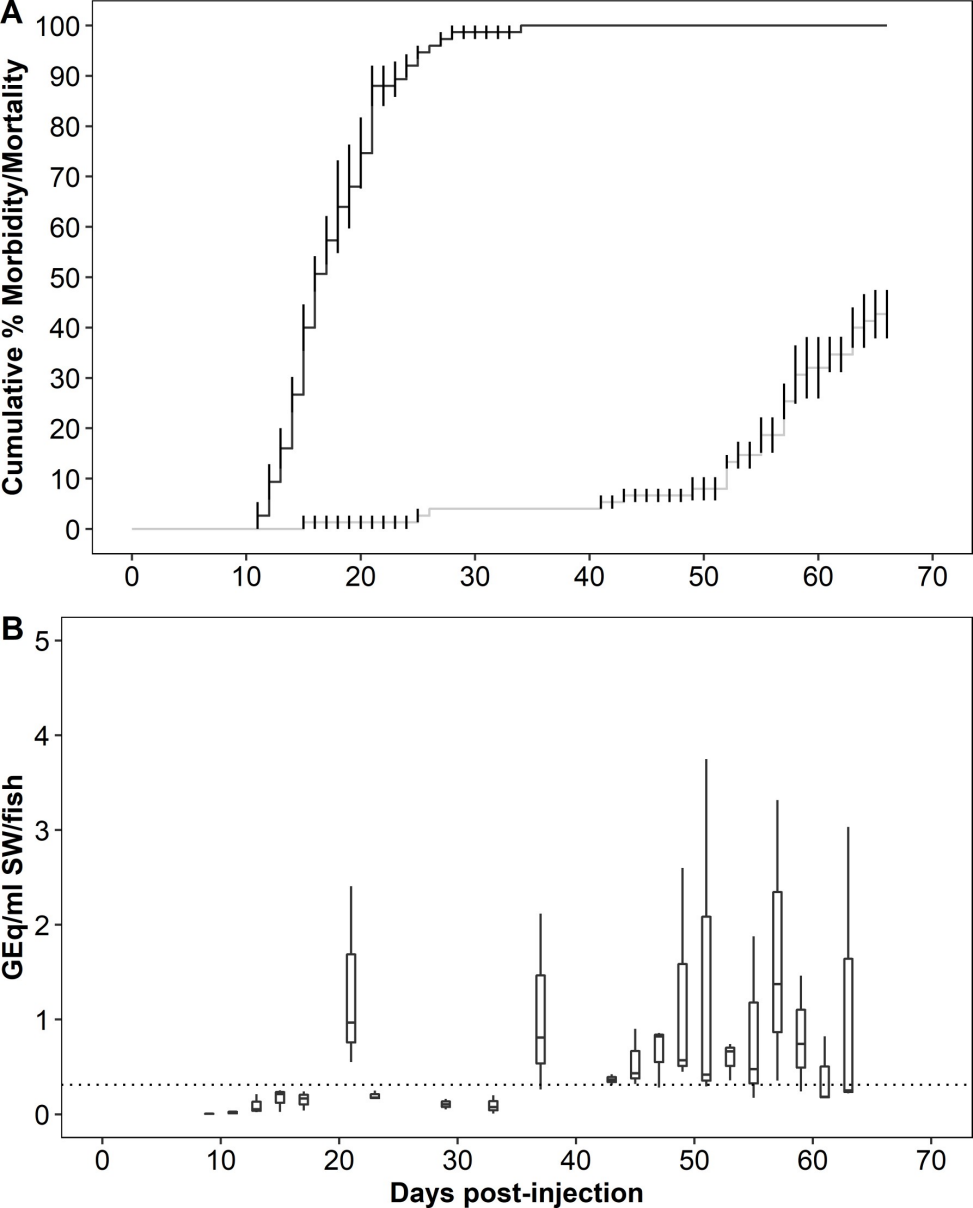
Mortality and bacterial burden in water during *Piscirickettsia salmonis* cohabitation challenge in chum salmon (Trial 2). (A) Cumulative mean percent morbidity and mortality for donor (black) and recipient (gray) populations, error bars denote standard error. (B) *P*. *salmonis* genome equivalents in tank water over the course of the trial. The dotted horizontal line denotes assay limit of quantification. See Fig 1 caption for box plot description.

In Trial 2 (chum cohabitation), the mean CMM among recipients was 42.7 ± 4.8 (Fig 2A) while the MDD were 17.5 (donor) and 56.4 (recipient). *Piscirickettsia salmonis* was detected in 100% of donor mortalities examined and in 83% of recipient mortalities. The bacterium was not detected in three mortalities that occurred in the recipient population between 18 and 26 dpi, and these fish were excluded from subsequent analysis. In mortalities, the median kidney burden in GEq/μg DNA was 4.2 (interquartile range, IQR = 0.4) in donors and 2.5 (IQR = 1.6) in recipients. The bacterium was detected in 8 of 17 surviving recipients with a median kidney burden of 1.8 (IQR = 0.9) GEq/μg DNA.

In Trial 3 (pink donors), mortalities began at 7 dpi and the MDD among the donor fish was 10.3. The 4 mortalities were not screened for the bacterium.

**Fig 3:**
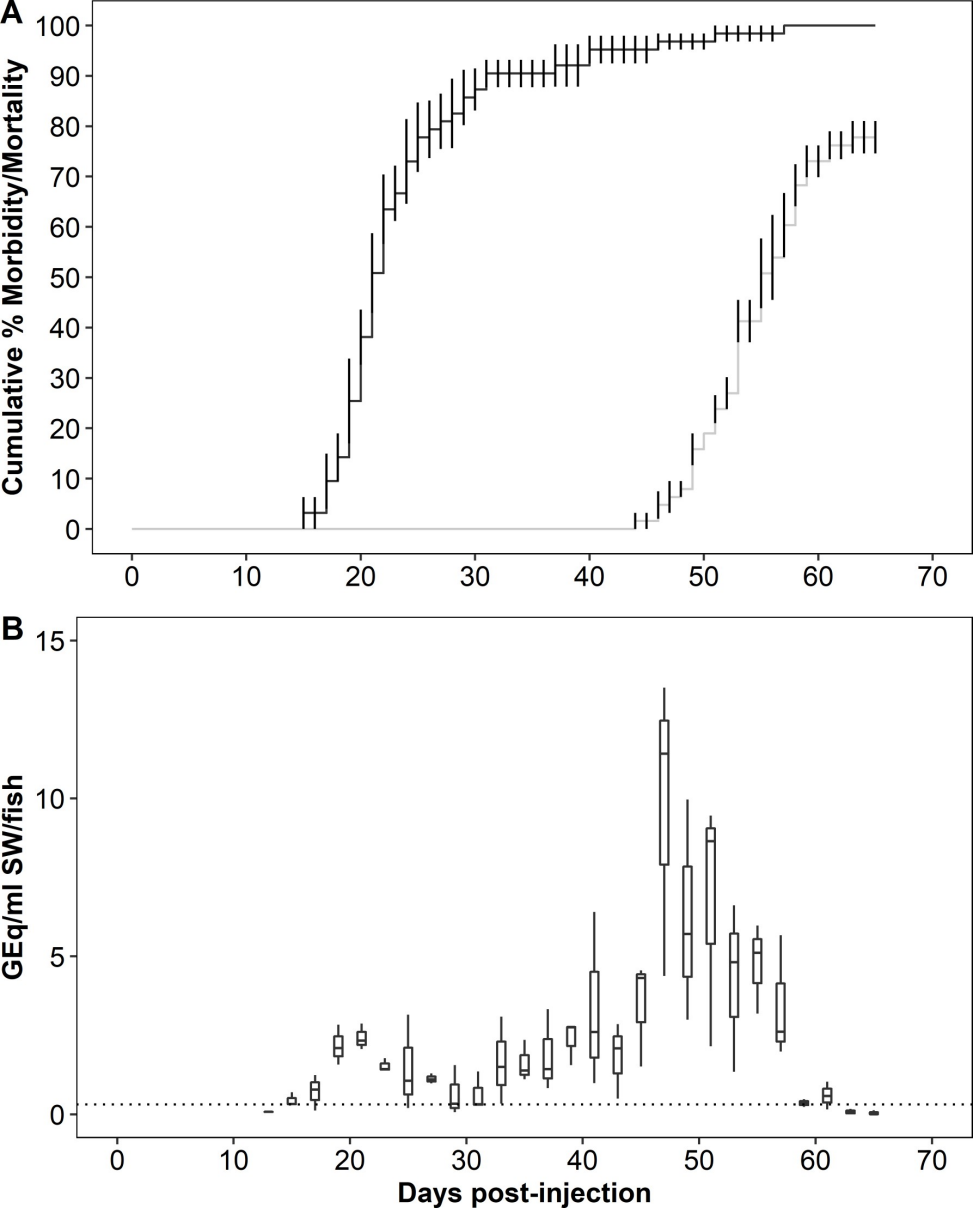
Mortality and bacterial burden in water during *Piscirickettsia salmonis* cohabitation challenge in Atlantic salmon (Trial 4). (A) Cumulative mean percent morbidity and mortality for donor (black) and recipient (gray) populations, error bars denote standard error. (B) *P*. *salmonis* genome equivalents in tank water over the course of the trial. The dotted horizontal line denotes assay limit of quantification. See Fig 1 caption for box plot description.

In Trial 4 (Atlantic cohabitation), the mean CMM among recipients was 77.8 ± 3.2 (Fig 3A), while the MDD were 23.9 (donor) and 53.7 (recipient). The bacterium was detected in 96% of mortalities examined. In the mortalities, the median kidney burden in GEq/μg DNA was 4.7 (IQR = 0.4) in donors (n = 8) and 3.3 (IQR = 2.4) in recipients (n = 15). The bacterium was detected in 2 of 14 surviving recipients with a median kidney burden of 1.6 (IQR = 0.5) GEq/μg DNA.

### Quantification of *P*. *salmonis* in seawater

The bacterial burden in water was estimated by measuring *P*. *salmonis* DNA in samples collected during the cohabitation trials. In Trial 1, bacterial DNA was detected in two peaks, the first beginning 15 dpi with a maximum at 17 dpi (8.8 GEq/mL/fish, IQR = 13.8), 3 d after the onset of mortality in the donor group (Fig 1B) and 2.7 d before the donor MDD. The maximum value of the second peak occurred 48 dpi (164.1 GEq/mL/fish, IQR = 165.0), 9 d after the onset of mortality in the recipient group and 2 d before the recipient MDD.

In Trial 2, bacterial DNA was first detected at 21 dpi (1.0 GEq/mL/fish; IQR = 0.93), 10 d after the onset of mortality in the donor group and 3.5 d after the donor MDD (Fig 2B). Bacterial DNA was also detected from 37 to 63 dpi, and the AUC during this interval was 10.3 GEq/mL/fish, with a maximum value at 57 dpi of 1.4 GEq/mL/fish (IQR = 1.5). The maximum occurred 16 d after the onset of mortality in the recipient group and 0.6 d after the recipient MDD.

In Trial 4, two peaks of waterborne bacterial DNA were detected between 15 and 29 dpi and between 31 and 61 dpi. The AUC during the first peak was 10.3 GEq/mL/fish, and that for the second peak was 53.3 GEq/mL/fish. Maximum median values at 21 dpi and 47 dpi were 2.3 GEq/mL /fish (IQR = 0.41) and 11.4 GEq/mL/fish (IQR = 4.6) (P = 0.04), respectively. In the first peak, the maximum occurred 6 d after the onset of mortality in the donor group and 2.9 d before the donor MDD (Fig 3B), whereas the maximum in the second peak occurred 3 d after the onset of mortality in the recipient group and 6.7 d before the recipient MDD.

### Individual fish sampling

In Trial 2, *P*. *salmonis* was detected in kidney from 100% of donors and from 50% of recipients (Table 2). In both the donor and recipient populations, the median bacterial burden was significantly greater at the second sampling point as compared to the first (*P* < 0.05). In Trial 3, the bacterium was detected in all sampled donors, and in Trial 4, the bacterium was detected in all sampled donors and recipients (Table 2). Differences in median bacterial burdens between sampling points for either donors or recipients were not statistically significant in Trials 3 and 4.

**Table 2:** *Piscirickettsia salmonis* burden in kidney and shedding rates in individual chum, pink, and Atlantic salmon.

Trial (Species)	Treatment	DPI	No. above LOQ	Median bacterial burden^1^^,^^3^	No. Shedding	Median shedding rate^2^
			(No. examined)			
2 (Chum)	Donor	10	10 (10)	2.8 (0.46)^a^	0	0 (0)
		15	10 (10)	3.8 (1.5)^b^	10	0.05 (0.1)
	Recipient	31	4 (10)	1.1 (0.18)^c^	4	0.03 (0.03)
		53	6 (10)	2.7 (1.1)^d^	6	0.13 (0.3)
3 (Pink)	Donor	8	10 (10)	3.8 (1.4)	0	0 (0)
		13	10 (10)	4.3 (0.9)	8	0.005 (0.03)
4 (Atlantic)	Donor	10	5 (5)	2.1 (0.9)	5	0 (0.006)
		17	12 (12)	2.0 (1.7)	10	0.008 (0.02)
	Recipient	38	5 (5)	1.5 (0.3)	4	0.001 (0.004)
		46	12 (12)	1.8 (0.7)	11	0.02 (0.04)

^1^Log_10_ Genome equivalents / μg DNA (interquartile range).

^2^Genome equivalents / mL seawater / g / min (interquartile range).

^3^Letters denote significant difference in bacterial burden between timepoints for each treatment.

**Fig 4:**
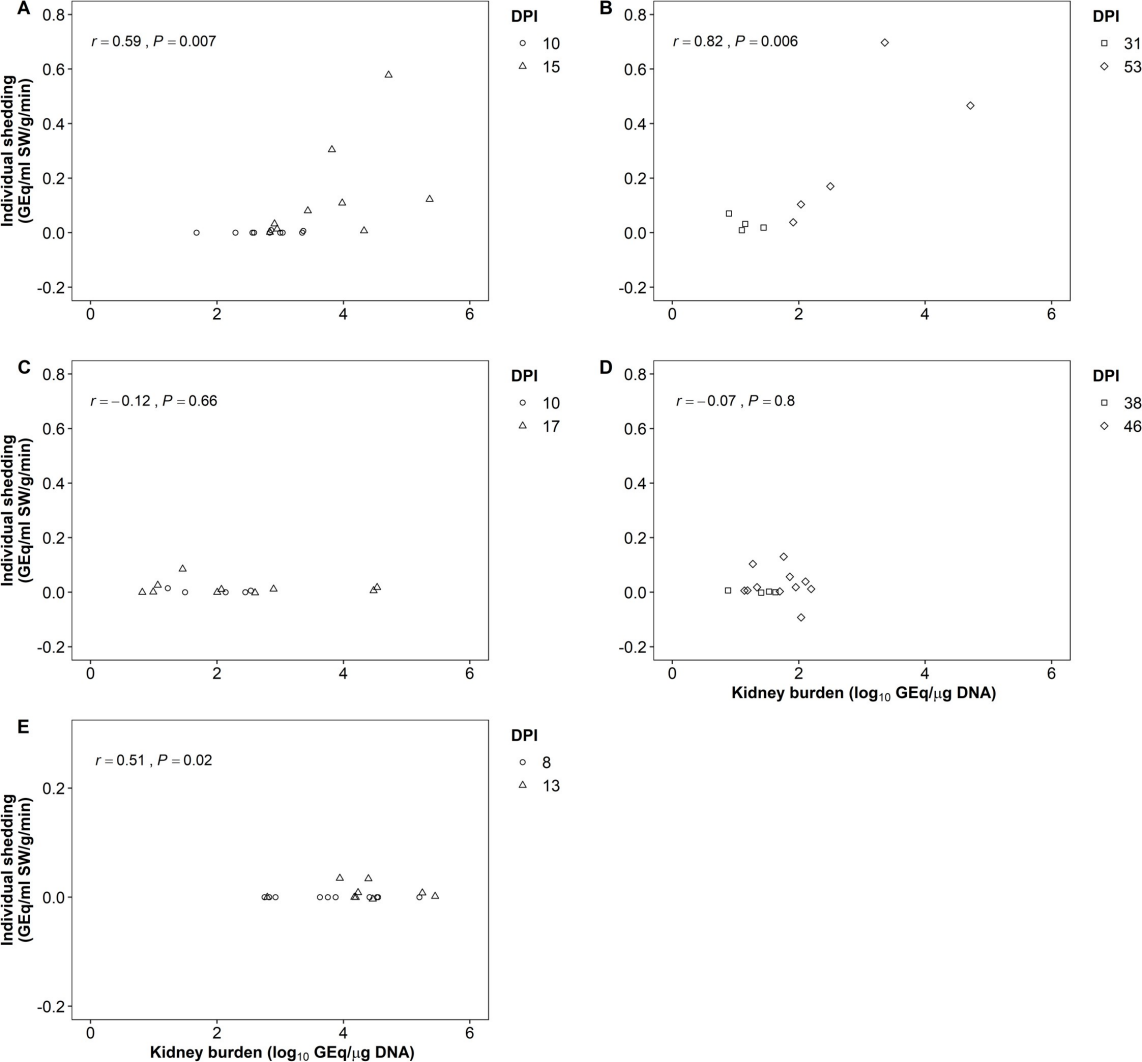
Relationship between individual kidney burden (log_10_ GEq/μg DNA) and shedding rate (GEq/mL SW/g/min) in (A) donor chum salmon at 10 and 15 dpi, (B) recipient chum salmon at 31 and 53 dpi, (C) donor Atlantic salmon at 10 and 17 dpi, (D) recipient Atlantic salmon at 38 and 46 dpi, and (E) donor pink salmon at 8 and 13 dpi.

Bacterial shedding rates from donor and recipient chum and Atlantic salmon and from donor pink salmon were measured (Table 2). No shedding was detected from any donor pink or chum salmon at the first sampling point, despite detection of *P*. *salmonis* in kidney tissue. There was a significant positive correlation between *P*. *salmonis* burden and shedding rate in kidney of donor and recipient chum salmon and in kidney of donor pink salmon, whereas this relationship was not statistically significant in Atlantic salmon (Fig 4).

**Fig 5:**
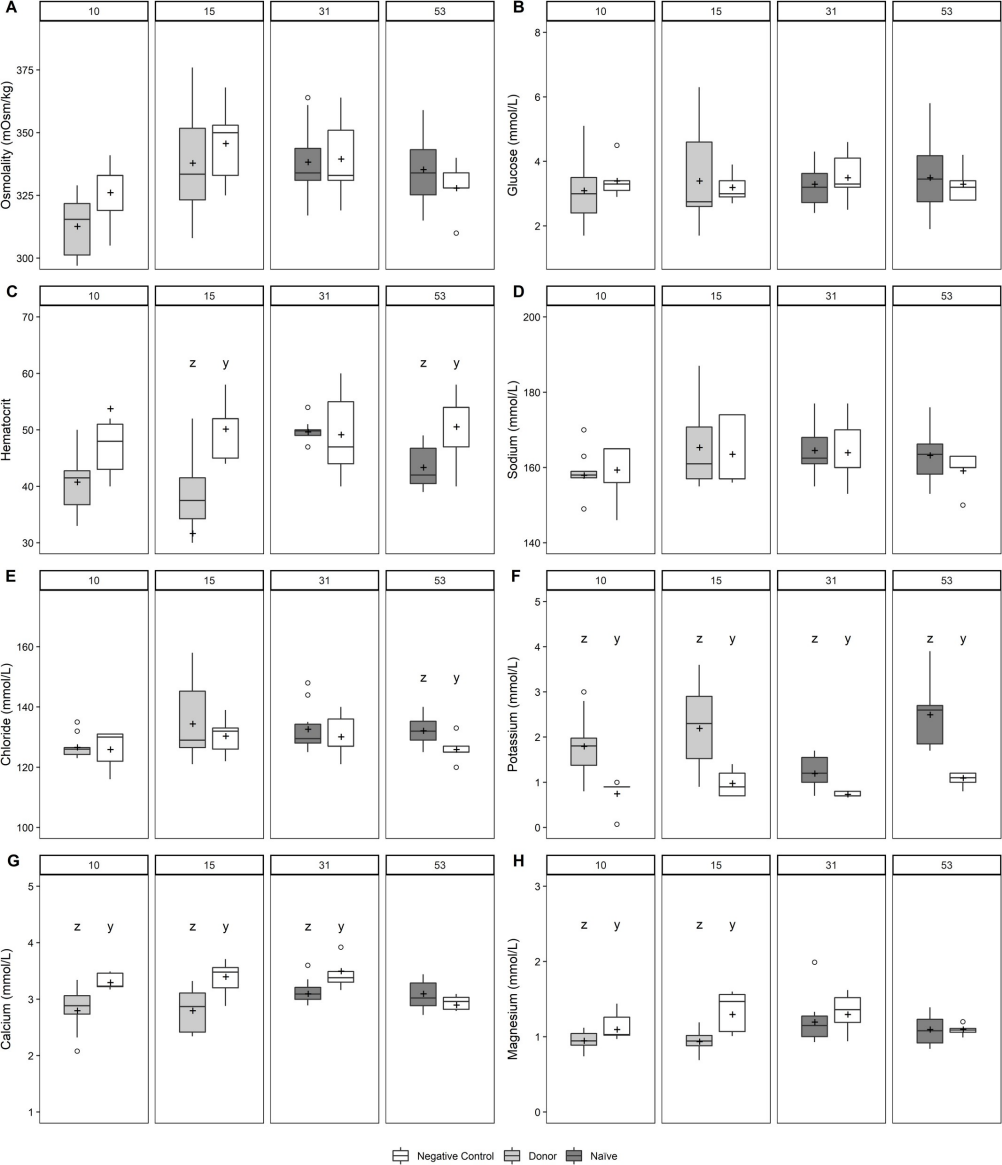
(A) Plasma osmolality, (B) glucose, (C) hematocrit, (D) sodium, (E) chloride, (F) potassium, (G) calcium, and (H) magnesium in donor and recipient chum salmon sampled at 10, 15, 31, and 53 d post-injection during *Piscirickettsia salmonis* cohabitation challenge. See caption for Fig 1 for box plot description. Letters above plots denote statistically significant differences between negative control and exposed fish within a sampling time (*P* ≤ 0.05).

**Fig 6:**
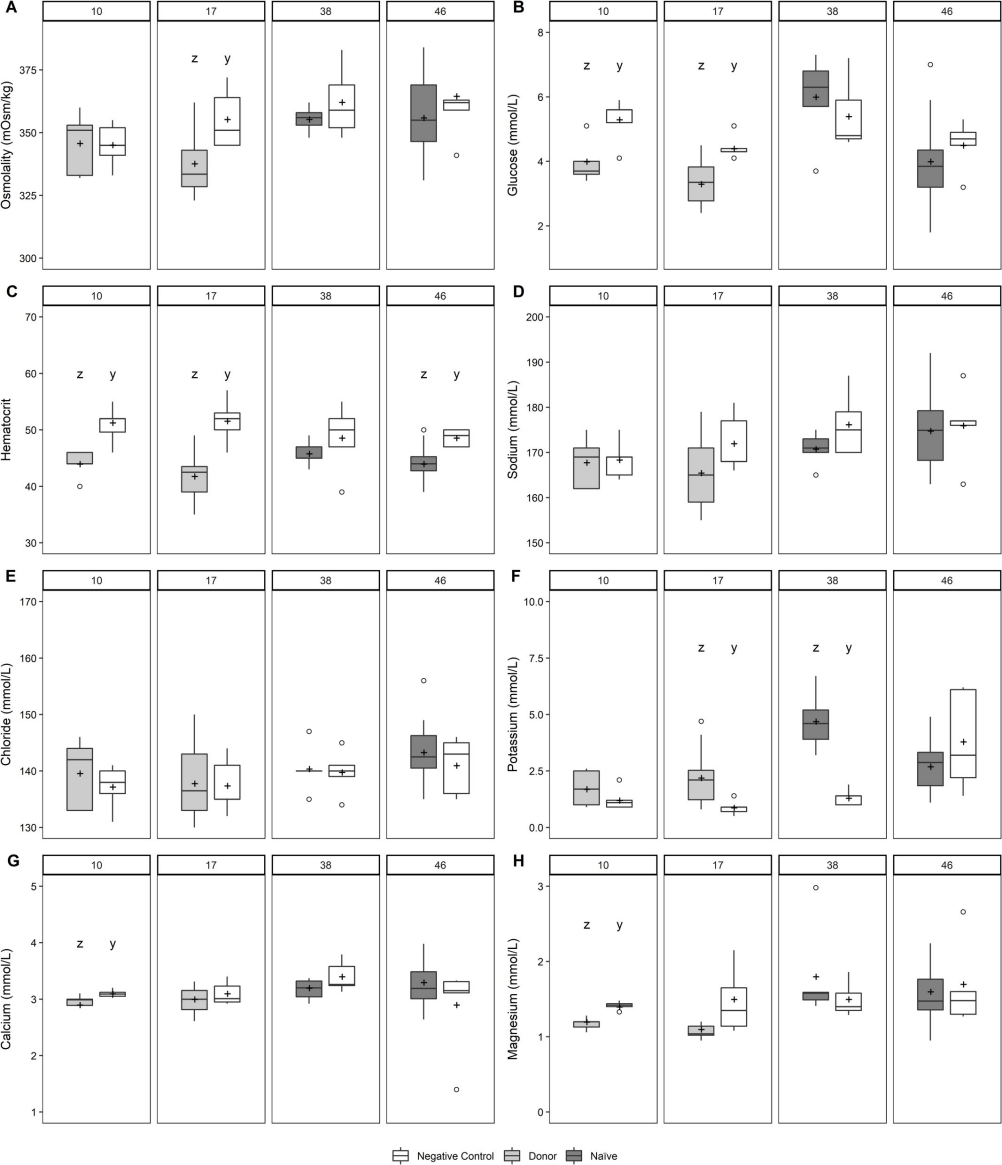
(A) Plasma osmolality, (B) glucose, (C) hematocrit, (D) sodium, (E) chloride, (F) potassium, (G) calcium, and (H) magnesium in donor and recipient Atlantic salmon sampled at 10, 17, 38, and 46 d post-injection during *Piscirickettsia salmonis* cohabitation challenge. See caption for Fig 1 for box plot description. Letters above plots denote statistically significant differences between negative control and exposed fish within a sampling time (*P* ≤ 0.05).

Mean hematocrit was significantly lower in infected chum and Atlantic salmon compared with the negative controls (Figs 5 and 6). During infections in both species, there were significant reductions in mean plasma Ca^++^ and Mg^++^, whereas mean plasma K^+^ was significantly elevated (Figs 5 and 6). Significant reductions in mean plasma osmolality and glucose were measured in infected Atlantic salmon (Fig 6), whereas mean plasma Cl^-^ was significantly elevated in infected chum salmon (Fig 5). Correlations between kidney bacterial burden and some plasma parameters were statistically significant. In infected Atlantic salmon, hematocrit, plasma Ca^++^ and Mg^++^ were negatively associated with bacterial burden, whereas in infected chum salmon, Na^+^ and Cl^-^ were positively associated (Table 3).

**Table 3:** Results of Pearson Product-Moment Correlation analysis of *P*. *salmonis* burden in kidney (log_10_ GEq/μg DNA) and physiology measurements from Trials 2 (chum salmon) and 4 (Atlantic salmon).

Factor	Trial 2 (Chum salmon)				Trial 4 (Atlantic salmon)			
	Donor		Recipient		Donor		Recipient	
	10 dpi	15 dpi	31 dpi	53 dpi	10 dpi	17 dpi	38 dpi	46 dpi
	(n = 10)	(n = 10)	(n = 4)	(n = 6)	(n = 5)	(n = 10)	(n = 4)	(n = 11)
Potassium	-0.01	0.34	NA	0.30	-0.31	0.57	NA	-0.22
Calcium	-0.40	-0.31	NA	0.20	0.15	-0.18	NA	**-0.77 (<0.01)**
Magnesium	-0.18	0.20	NA	0.39	-0.60	0.13	NA	**-0.65 (0.03)**
Osmolality	-0.12	0.51	NA	0.56	0.04	0.04	NA	-0.52
Sodium	-0.22	**0.78 (<0.01)**	NA	0.39	0.10	0.02	NA	-0.42
Chloride	-0.06	**0.68 (0.03)**	NA	0.89	0.09	0.32	NA	-0.10
Hematocrit	0.05	0.49	NA	0.82	-0.76	**-0.79 (<0.01)**	NA	-0.18
Glucose	**-0.86 (0.02)**	0.43	NA	0.29	-0.57	-0.48	NA	-0.14

If the correlation was statistically significant (*P* < 0.05), *r* values are bolded and the *P*-value is given in parentheses. NA denotes analysis not done due to small sample size.

## Discussion

Injection and cohabitation laboratory exposure trials were used to better understand the transmission of *Piscirickettsia salmonis* and risk factors associated with SRS. To do so, bacterial shedding was measured in association with mortality, tissue bacterial burdens and plasma physiological parameters in three salmonid species. Presence of the bacterium in dead, moribund and surviving recipients confirmed the occurrence of transmission from donors which, together with the temporally-variable evidence of bacterial DNA in water, permitted the characterization of bacterial shedding patterns. In these trials, all injected donor fish died in contrast to the reduced cumulative morbidity and mortality (CMM) observed among cohabitant recipients (chum salmon, 43%; pink salmon, 67%; Atlantic salmon, 78%). This dichotomy suggested pathogenesis differed both in relation to the route of exposure to the bacterium and among species, warranting a closer examination of bacterial shedding from the donor and recipient salmon.

The bimodal pattern of waterborne bacterial DNA measured during all cohabitation trials was interpreted as shedding of *P*. *salmonis* from the donor and recipient populations, respectively. In addition, in all trials relatively fewer bacteria were shed during infection in donors. Both of these patterns were most obvious in Trial 4 in which sampling frequency was high throughout. However, since kidney bacterial burdens were equal to or greater in donors than in recipients, reduced bacterial burdens in donors was not the likely explanation of lower overall shedding from this group. Rather, reduced levels of donor shedding may have been related to the relative rapidity with which injection-initiated infections caused morbidity and death, resulting in a shorter interval of shedding from progressively fewer donor fish. Coincidental shedding from late surviving donors with early shedding from recipients was suggested by the gradual increase in waterborne bacterial DNA during the second peak in Trial 4 (see Fig 3A). Early and transient shedding from recipient chum salmon may also explain the elevated waterborne bacterial DNA observed at 37 dpi in Trial 2. In all trials, maximum shedding coincided approximately with mean days-to-death (MDD) in the donor and recipient populations. In pink and Atlantic salmon, maximum shedding preceded the MDD, similar to our previous report of Atlantic and sockeye salmon following immersion exposure to *P*. *salmonis* [14]. In other work, maximum shedding of infectious hematopoietic necrosis virus precedes peak mortality in rainbow trout (*O*. *mykiss*) [24] and in Atlantic salmon [25]. These patterns are consistent with the intuitive expectation that during an infection, maximum pathogen shedding occurs shortly before mortality and that the subsequent decline in shedding (see Fig 3B) coincides with mortality among shedders and/or recovery from the infection. That maximum shedding of *P*. *salmonis* from chum salmon appeared to follow the MDD was possibly an artefact related to the sampling design. However, the apparent similarity of this pattern to an earlier report in which Chinook salmon held at 8°C but not at 12 and 15°C, shed *Renibacterium salmoninarum* at a maximum rate after the MDD [26] was notable. While it is possible that the later and reduced morbidity and mortality among recipient chum relative to pink salmon and Atlantic salmon may have altered the timing of bacterial shedding, more research is required to better understand this apparently counter-intuitive pattern of *P*. *salmonis* shedding by chum salmon.

Variations in the overall magnitude of waterborne DNA were observed among trials indicating differential shedding of *P*. *salmonis* from both recipient and donor salmon. Since the four trials were conducted over a period of 10 months with fish of different species, sizes and sampling regimes, it is premature to conclude that the shedding patterns reflected differences in susceptibility to the bacterium among the species. Nevertheless, at a median value of 8.8 GEq/mL/fish, maximum shedding from donor pink salmon was nearly 4-times greater than the maximum values from donors in the other species. Although our confidence in the shedding estimates for recipient pink salmon was limited by the reduced number of water samples collected during the latter part of the trial and some caution is required in their interpretation, the maximum median shedding rate of 164.1 GEq/mL/fish at 48 dpi that was at least 14-times higher than that from recipients in the other species. In the other species, maximum *P*. *salmonis* shedding from donor Atlantic salmon was approximately double that of chum salmon donors. Also, the maximum shedding from recipient Atlantic salmon was 10-fold higher and the total shed bacteria was 5-fold higher relative to chum salmon recipients. Given that waterborne bacterial burdens were normalized to fish number rather than to biomass and that the mean sizes of pink and Atlantic were approximately 3- to 4-fold larger than that of chum salmon (see Table 1), fish size must be considered as a factor in explaining the reduced shedding rate from the chum salmon. In earlier work [14], reduced CMM and bacterial shedding, together with reduced prevalence and magnitude of bacterial tissue burdens in survivors were used to conclude a low susceptibility to *P*. *salmonis* in sockeye salmon. In this context, the infections in chum salmon recipients were seemingly contradictory in eliciting a lower CMM and a higher prevalence of kidney infection relative to Atlantic salmon. Furthermore, while there was a direct relationship between individual size and shedding rate in Atlantic salmon, no such relationship was evident in pink or chum salmon, and a range of shedding rates occurred among members of the latter two species, regardless of size (S1 Fig). These data support the hypothesis that shedding of *P*. *salmonis* was greatest in pink salmon and least in chum salmon. Further research using concurrent trials and size-matched salmon is required to test this hypothesis.

An estimate of bacterial shedding from individual fish was used to assist in the interpretation of shedding patterns observed from water samples collected during the cohabitation trials. There were two times (see Table 2) when shedding was observed from all sampled fish and in both occasions, these infections had been initiated by injection. In all other sampling events, the shedding of *P*. *salmonis* occurred in 0% to 92% of fish sampled among the three species. The absence of detectable shedding was noteworthy in the initial samples from injected pink and chum salmon in that kidney bacterial infections were detected in all these fish, suggesting that infections in this organ do not contribute directly to shedding. Variations in individual shedding rates, including no detectable shedding in fish with severe infections, have been reported for other fish pathogens [24, 26–28]. Chinook salmon injected with a low dose of *R*. *salmoninarum* did not shed detectable levels of the bacterium, whereas shedding from those injected with a high dose was detectable by 12 dpi, suggesting that exposure dose influences shedding of this organism [28].

In the present study, alignment of the timing of individual sample events with the onset of clinical disease and the mid-point of the CMM curve varied among the trials. Therefore, comparisons of absolute individual shedding data among trials were considered to be of limited value. The focus instead was on whether bacterial burdens in kidney were predictive of bacterial shedding rate and of plasma physiological parameters in Atlantic salmon and chum salmon. The original hypothesis was of no relationship between tissue bacterial burden and shedding rate, and indeed this was the case with Atlantic salmon. However, in chum and pink salmon, shedding rates were correlated to bacterial kidney burdens suggesting the greater importance of bacterial shedding via the urinary system in these species compared to the Atlantic salmon. Tissue damage resulting from infection may play a role in pathogen shedding [24]. Necrosis of kidneys due to infection can inhibit fish urinary activity, possibly decreasing pathogen shedding via urine which is relevant as *P*. *salmonis* may be excreted via feces, bile, and urine [2]. In addition, systemic infections with this bacterium result in necrosis in kidney, liver, and intestine [21, 29], and the most severe liver pathology occurs in Atlantic salmon carrying the heaviest burden of *P*. *salmonis* [14]. Additional research is required to understand patterns of tissue damage and *P*. *salmonis* shedding during infections that are characterized by the sequential involvement of various organ systems.

Values of hematocrit and several plasma ions changed in association with the timing or severity of *P*. *salmonis* infection in Atlantic salmon and chum salmon. In donors and recipients of both species, declining hematocrit values coincided with disease progression, but correlated with kidney bacterial burden only in donor Atlantic salmon at 17 dpi. Low hematocrit values have been reported in coho salmon (*O*. *kisutch*) during SRS outbreaks [29]. While elevated plasma potassium was associated with the infection in both species, there was no evidence that the concentration of this plasma ion correlated with kidney bacterial burden. Hyperkalemia results from the breakdown of cells in blood and other tissues, and has been previously reported in salmon infected with *R*. *salmoninarum* [30]. Hyperkalemia may also result from decreased potassium excretion due to renal tissue damage from stress or physical injury [30, 31]. The reduced levels of plasma Ca^++^ and Mg^++^, indicative of damage to kidney and intestine [32], had a longer duration in donor chum salmon. In Atlantic salmon, although reductions of these divalent cations were observed in the earliest donor samples, they correlated with kidney bacterial burdens only in recipients sampled at the onset of clinical disease, possibly the result of tissue damage in this species. Previous studies of Atlantic salmon infected with *P*. *salmonis* have reported serum biomarkers indicative of kidney and liver damage [33]. Whereas a decline in plasma glucose was consistently observed in donor Atlantic salmon, a negative correlation with kidney bacterial burden was evident only in donor chum salmon. Hypoglycemia previously reported in Atlantic salmon infected with *Aeromonas salmonicida* may have been related to hemodilution, loss via cutaneous lesions, or utilization by the infectious agent [34]. Our inconsistent evidence for osmotic perturbation in chum salmon and Atlantic salmon does not support hemodilution as an explanation for the hypoglycemia or reduced hematocrit during *P*. *salmonis* infections.

In conclusion, the utility of cohabitation as a reproducible model of laboratory infection with *P*. *salmonis* as described earlier [11, 33] was confirmed in the current study in two species of Pacific salmon and in Atlantic salmon. Despite variation among trials, the demonstrated coincidence of bacterial shedding with mortality will inform pathogen transmission models. Furthermore, within species there was evidence in all trials that the magnitude of bacterial shedding varied among individuals, but not consistently as a function of fish weight. However unlike in pink salmon and chum salmon, no demonstrable correlation between *P*. *salmonis* shedding and kidney bacterial burden in Atlantic salmon was evident. Our findings supported none of the null hypotheses and indicated a dependency of bacterial shedding on species-specific patterns of pathogenesis. The recognition of a suite of plasma physiological parameters shown to be associated with SRS laid the groundwork for improved definition of biomarkers of pathogenesis and bacterial shedding.

## Supporting information

S1 Fig Regression plots of *Piscirickettsia salmonis* shedding and fish weight in pink salmon (*Oncorhynchus gorbuscha*), chum salmon (*O*. *keta*) and Atlantic salmon (*Salmo salar*). (DOCX)
