# Supplementary material for: Piscirickettsia salmonis shedding and tissue burden, and hematological responses during cohabitation infections in chum Oncorhynchus keta, pink O. gorbuscha and Atlantic salmon Salmo salar
Source: PLoS One. 2021 Mar 5;16(3):e0248098. doi: 10.1371/journal.pone.0248098 (PMC7935282; doi:10.1371/journal.pone.0248098)
Supplement: S1 Fig — (DOCX) [file pone.0248098.s001.docx]

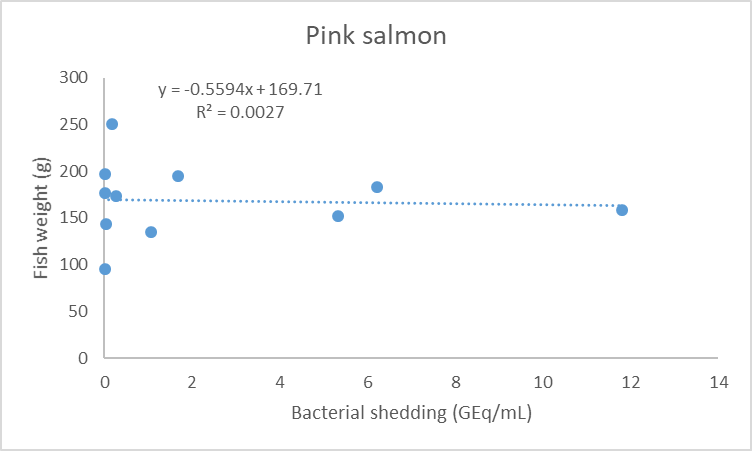


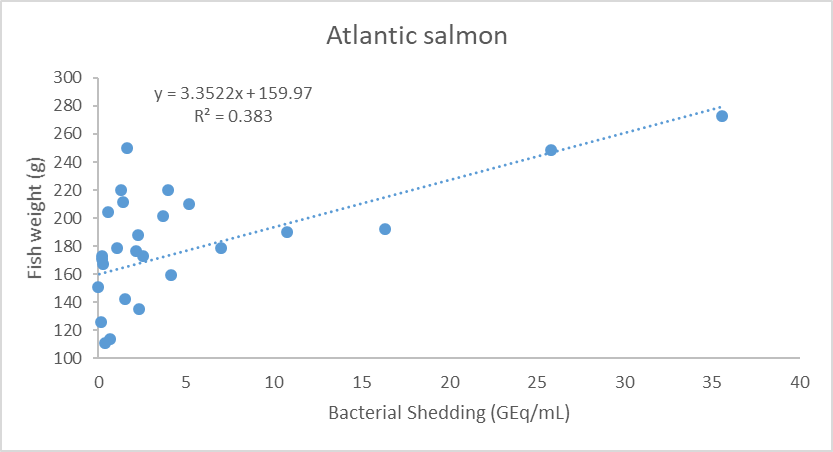


S1 Fig. Regression plots of *Piscirickettsia salmonis* shedding and fish weight in pink salmon (*Oncorhynchus gorbuscha*), chum salmon (*O. keta*) and Atlantic salmon (*Salmo salar*)
